# Supplementary material for: Interstellar Benzene Formation Mechanisms via Acetylene Cyclotrimerization Catalyzed by Fe+ Attached to Water Ice Clusters: Quantum Chemistry Calculation Study
Source: Molecules. 2022 Nov 11;27(22):7767. doi: 10.3390/molecules27227767 (PMC9693163; doi:10.3390/molecules27227767)
Supplement: Supplementary file 1 [file molecules-27-07767-s001.zip › molecules-1998847-supplementary.pdf]

Interstellar Benzene Formation Mechanisms via Acetylene Cyclotrimerization Catalyzed by Fe^{+} Attached to Water Ice Clusters: Quantum Chemistry Calculation Study

Tatsuhiro Murakami[1,2] and Toshiyuki Takayanagi [1,*]

[1] Department of Chemistry, Saitama University, Shimo-Okubo 255, Sakura-ku, Saitama City, Saitama 338-8570, Japan
[2] Department of Materials & Life Sciences, Faculty of Science & Technology, Sophia University, 7-1 Kioicho, Chiyoda-ku, Tokyo, 102-8554, Japan
* Correspondence: murakamit@mail.saitama-u.ac.jp (T.M.); tako@mail.saitama-u.ac.jp (T.T.); Tel.: +81-48-858-9113 (T.M & T.T.)

Supplementary Material

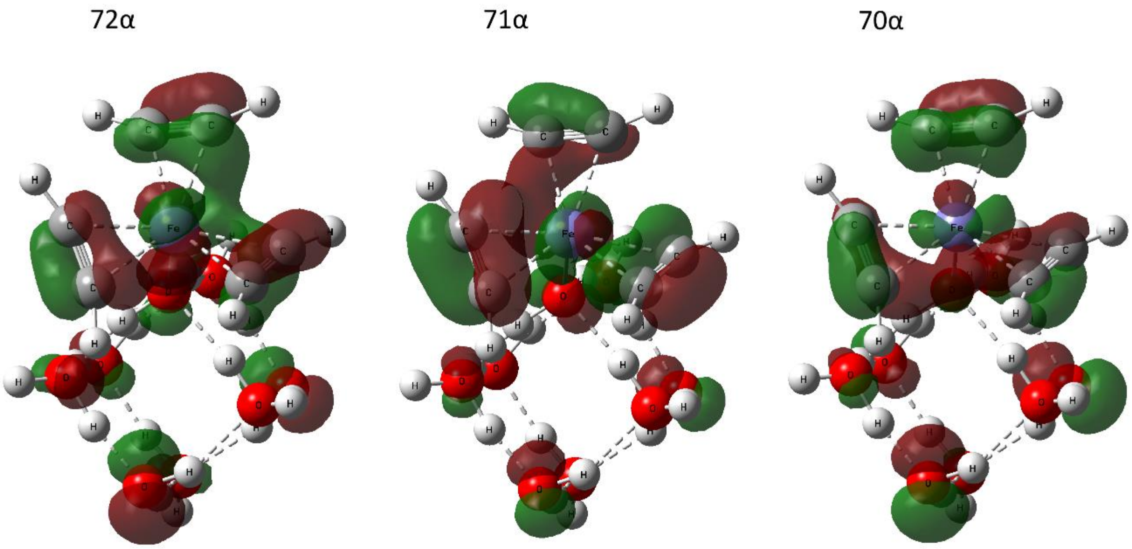

Figure S1. The three Kohn-Sham orbitals associated with the contribution of the d-π bonding interactions in the $(C_2H_2)_3Fe^+(H_2O)_8$ reactant complex

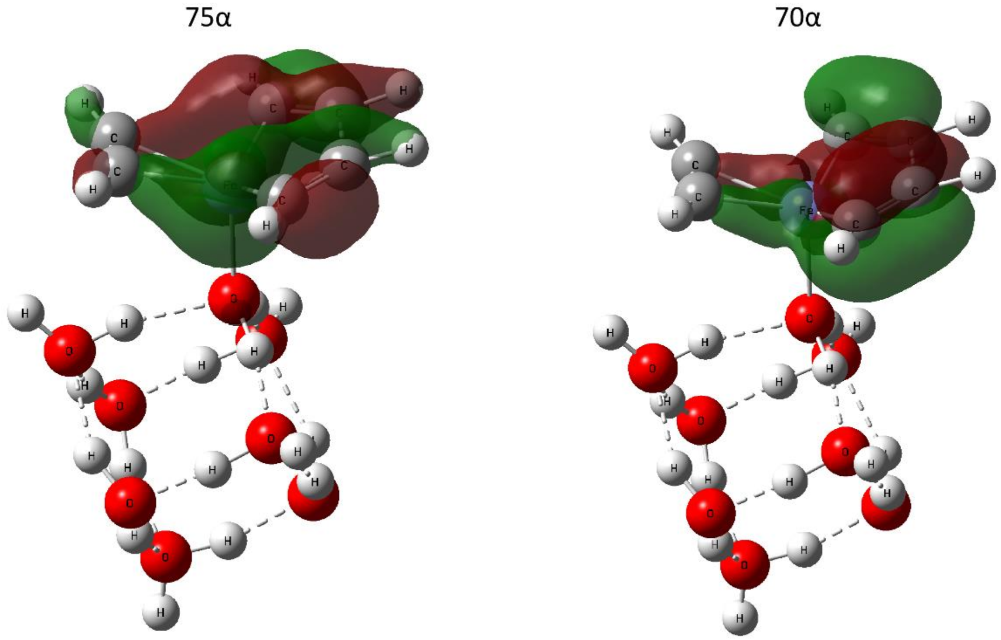

Figure S2. The two Kohn-Sham orbitals associated with the two CC σ-bonding interaction at the TS_{w8} structure.

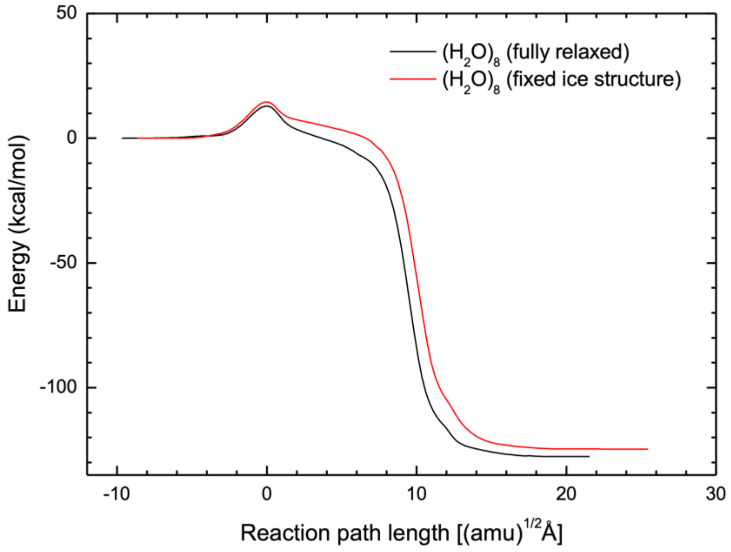

Figure S3. Comparison of the potential energy curves at fully relaxed coordinate (black line) and at fixed ice structure (red line) along IRC pathway.

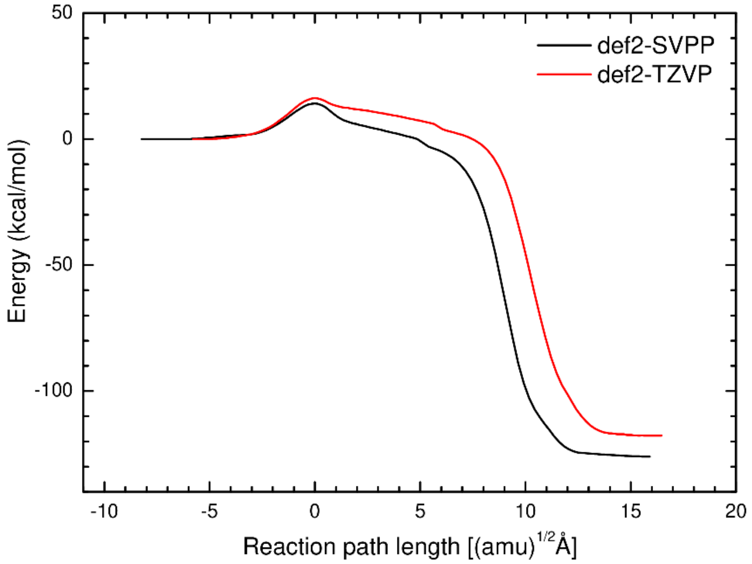

Figure S4. Comparison of the potential energy profiles for the acetylene cyclotrimerization reactions catalyzed by the $Fe^{+}(H_2O)$ complex calculated using the def2-SVPP (black line) and def2-TZVP (red line) basis sets.

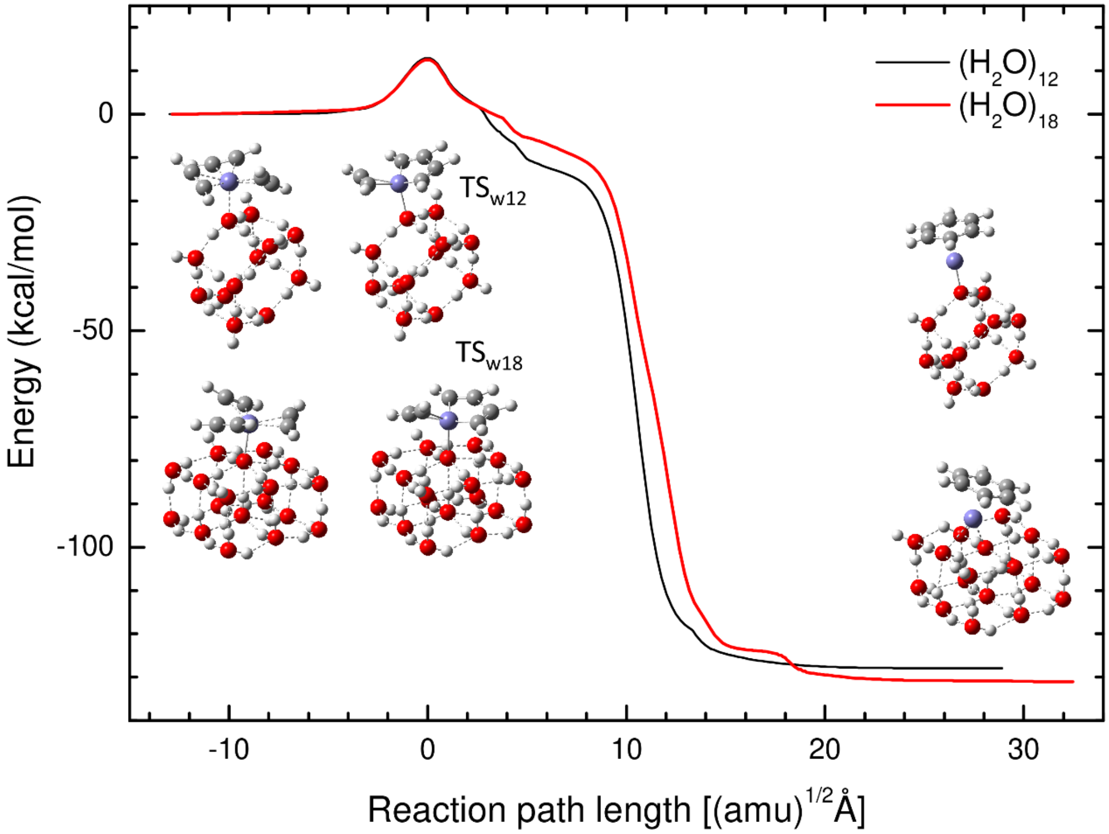

Figure S5. Comparison of the potential energy profiles for the acetylene cyclotrimerization reactions catalyzed by the $Fe^{+}(H_2O)_{12}$ (black line) and $Fe^{+}(H_2O)_{18}$ (red line) complexes calculated at the B3LYP(D).def2-SVPP level.

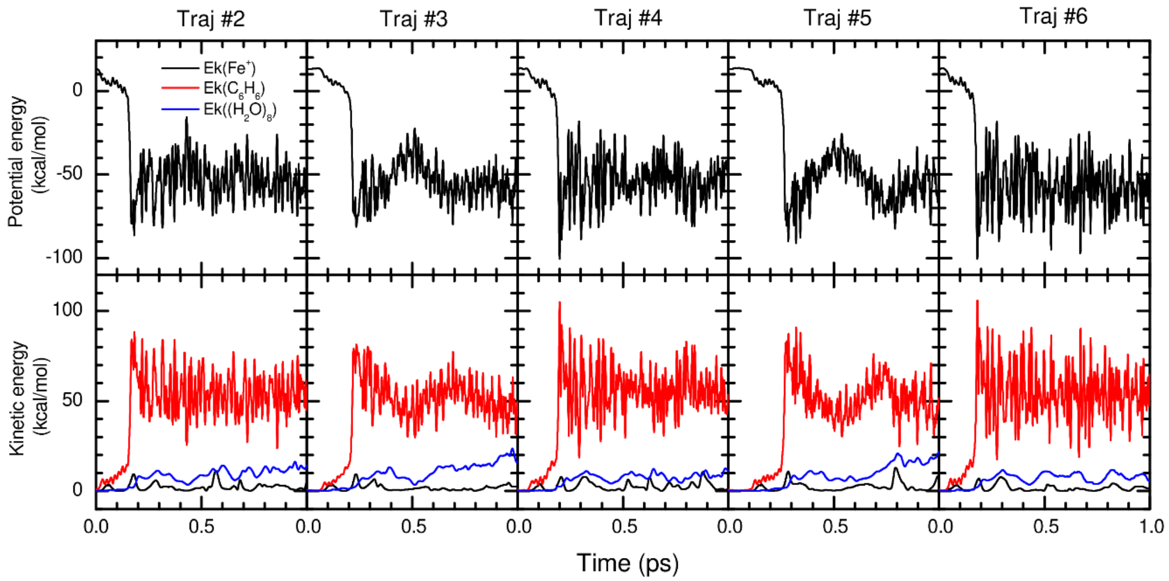

Figure S6. Potential energy and atomic kinetic energies obtained from the five BOMD trajectories starting from the transition state structure with an excess energy of 1 kcal/mol plotted as a function of simulation time. All the trajectories show the production of the C_6H_6-$Fe^+(H_2O)_8$ complex. Potential energy profiles are plotted in the upper panels, while the atomic kinetic energies are plotted in the lower panels. Black, red, and blue curves in the lower panels indicate the atomic kinetic energies in the Fe^+, C_6H_6, and $(H_2O)_8$ moieties, respectively.

Cartesian coordinates of stationary point geometries (in unit of Å) obtained from the B3LYP/def2-SVPP (with empiricaldispersion=GD3BJ, int=ultrafine options) level of theory. Total energies and zero-point vibrational energies are also shown.

****** *n* = 1 cluster **

$(C_2H_2)_2$-Fe^+(H_2O)

Fe	-0.95393754	-2.24410700	0.17182113
C	-1.23686707	-0.88867244	-1.31658457
C	-1.60318108	-3.65886295	1.57425656
C	-1.71519463	-2.52847937	2.08262518
C	-2.34953275	-1.18602247	-0.81290687
H	-0.58939224	-0.34104919	-1.99841313
H	-1.63909094	-4.73761730	1.45379724
H	-1.94258811	-1.73110763	2.78455455
H	-3.42505073	-1.19003991	-0.65449657
H	1.46430665	-3.56142398	0.11651946
H	1.22712425	-2.97293382	-1.33244619
O	0.84585698	-3.04947967	-0.43803648

ENERGY = -1494.32466289
ZPVE = 0.081032332009

Reactant

Fe	-0.77036635	-1.65192325	0.51900216
C	0.51585942	-0.51640976	1.78663799
C	-1.27877905	-0.92655720	-1.39733014
C	-1.81580499	-3.29294586	1.35805266
C	-1.41870024	-2.59722739	2.30671176
C	0.10397232	0.24418482	0.89667404
C	-2.25037398	-1.57858564	-0.97641200
H	1.04457194	-0.85845621	2.66829848
H	-0.66429147	-0.31927021	-2.05197224
H	-2.29095935	-4.09863495	0.81124893
H	-1.26583038	-2.23715936	3.31658507
H	-0.06872088	1.13794273	0.31011233
H	-3.24003788	-2.01735209	-0.92707659
H	1.50278394	-3.29081288	0.19266693
H	0.77066421	-3.23863291	-1.20479073
O	0.80414832	-2.84069399	-0.31599550

ENERGY = -1571.62616848
ZPVE = 0.109654962612

Transition state (TS_{w1})

Fe	-0.72217608	-1.83297119	0.56540020
C	0.19562321	-0.40977977	1.54319736
C	-1.27810502	-0.41367461	-0.96033044
C	-2.05161793	-3.08197697	1.38040358

C	-1.28686550	-2.66423932	2.29244912
C	-0.07768633	0.22148101	0.44981053
C	-1.92345360	-1.53247216	-0.94837367
H	0.68411260	-0.22307327	2.49875109
H	-1.22917409	0.47890889	-1.58218749
H	-2.88487937	-3.65205169	0.98060302
H	-0.92733606	-2.58312757	3.31383352
H	0.12907736	1.19701562	0.01230324
H	-2.72013520	-2.02739132	-1.50231245
H	1.75285365	-2.97753901	0.08439639
H	0.77670268	-3.51586437	-1.04419010
O	0.85740532	-2.90000896	-0.29291650

ENERGY = -1571.60366018
ZPVE = 0.111572915872

Product

Fe	-0.29880874	-2.26102205	0.29481743
C	-0.63333004	-0.45810568	1.86398101
C	-1.49378470	-0.29089112	-0.42268356
C	-2.35501539	-2.01329355	1.08685676
C	-1.51576231	-1.51978225	2.12220164
C	-0.57736505	0.11935314	0.57378031
C	-2.37838375	-1.35241453	-0.17118469
H	0.03305252	-0.09829170	2.65242531
H	-1.49203852	0.19800607	-1.40051519
H	-3.05055888	-2.83122688	1.28877812
H	-1.54603632	-1.97817054	3.11404641
H	0.12571299	0.93019011	0.36851851
H	-3.07380678	-1.68172089	-0.94754093
H	2.17857535	-2.82608882	-0.67691501
H	1.41727746	-4.19654954	-0.49135156
O	1.33844511	-3.22942723	-0.39105566

ENERGY = -1571.82686666
ZPVE = 0.124594813835

****** n = 8 cluster **

$(C_2H_2)_2$-$Fe^{+}(H_2O)_8$

Fe	-1.02685761	-2.11415418	0.47985235
C	-2.30098832	-2.46693373	-1.02585707
C	-1.08050369	-1.13609851	2.33303198
C	-0.09928360	-0.68395863	1.71925892
C	-2.55360161	-1.33349257	-0.52763067
H	-2.48650753	-3.27277606	-1.73289944
H	-1.83512417	-1.28639956	3.09869519
H	0.78956796	-0.11711713	1.46381969
H	-3.07498587	-0.38545416	-0.41930556
H	4.22940077	-4.97207312	-1.32814235

H	5.00640348	-6.38684976	-1.56119189
H	3.67770440	-3.20321168	-0.35184615
H	3.10858328	-3.28029653	-1.81074813
H	2.89843746	-4.48138211	1.27372803
H	2.97499992	-3.01288033	2.04201238
H	1.33286585	-3.32219198	0.60286340
H	0.74775377	-3.41765651	-0.95374130
H	1.48758056	-4.67348219	-2.41689007
H	1.00978006	-3.24249635	-3.12602758
H	2.66853990	-6.24950591	-2.05366938
H	1.18417158	-6.40188508	-1.51208405
H	3.58128195	-6.12630722	0.34451522
H	2.11682023	-6.27042647	0.93943369
H	0.24253840	-5.20793411	0.20222000
H	-0.30512629	-6.67882752	0.48694296
O	4.16874773	-5.99308314	-1.26162935
O	3.92374201	-3.43414805	-1.27796108
O	2.71060894	-3.43877262	1.20842292
O	0.45969235	-3.37572836	0.04441719
O	1.36260910	-3.62530636	-2.30445152
O	1.71031253	-6.09506132	-2.28710214
O	3.01491788	-5.91644436	1.13906327
O	0.45902811	-6.17252362	0.16015153

ENERGY = -2028.95068501
ZPVE = 0.258033695729

Reactant

Fe	-0.64914436	-1.69406553	0.48965601
C	0.12670423	-0.01943600	1.52837784
C	-1.72283899	-1.15024343	-1.24226416
C	-1.80856739	-2.95200246	1.75533983
C	-0.83396513	-2.57342654	2.42616822
C	0.35469240	0.15324183	0.31898513
C	-1.91286803	-2.36645857	-1.06764082
H	0.08208792	0.17574981	2.59249713
H	-1.79691635	-0.15862147	-1.67157713
H	-2.74814466	-3.42527372	1.49895606
H	-0.15835396	-2.45562898	3.26449617
H	0.70908834	0.60884525	-0.59770890
H	-2.27895698	-3.37246566	-1.23221770
H	3.82114872	-5.56014234	-1.61843585
H	4.31215149	-7.10603720	-1.45737703
H	3.81449003	-3.49660314	-1.32375675
H	2.83795900	-3.93910060	-2.46890902
H	3.46095722	-3.89874747	0.81752252
H	3.96935426	-2.32044607	0.83949095
H	1.87882265	-2.75113988	0.13211614
H	0.87400579	-3.23111081	-1.09362428
H	0.93688489	-5.03699982	-2.10229748

H	0.48519677	-3.88099953	-3.21132990
H	1.97048283	-6.62057756	-1.40411899
H	0.71254031	-6.25979159	-0.51477019
H	3.60779416	-5.88856541	0.48325529
H	2.39836160	-5.49663643	1.42253544
H	0.59349881	-4.40685371	0.72326491
H	-0.07918529	-5.41069053	1.75849540
O	3.66054675	-6.45548188	-1.14367763
O	3.74758898	-4.08080127	-2.11612357
O	3.38598317	-2.94546802	0.37632138
O	0.89183199	-2.89818126	-0.12060543
O	0.99527824	-4.02686181	-2.39687123
O	1.01079994	-6.35015778	-1.45003498
O	3.34874766	-5.30136756	1.24647162
O	0.60546034	-5.33738787	1.07236268

ENERGY = -2106.25041081
ZPVE = 0.288260679228

Transition state (TS_{w8})

Fe	-0.63704751	-1.84128134	0.52714382
C	0.29025200	-0.34948781	1.40180177
C	-1.33980625	-0.48639526	-1.01503976
C	-1.94286482	-3.04058436	1.48049584
C	-1.14160297	-2.57308134	2.33326055
C	-0.08348033	0.24654245	0.32200289
C	-1.91604210	-1.63712373	-0.94697109
H	0.83577721	-0.13352525	2.31889722
H	-1.37009446	0.40046158	-1.64503261
H	-2.79719459	-3.62082823	1.14594821
H	-0.74324324	-2.42248852	3.33180898
H	0.03475449	1.21981992	-0.15031102
H	-2.70261074	-2.19881440	-1.44808284
H	3.87275069	-5.57785903	-1.62068603
H	4.47432440	-7.06962310	-1.35323687
H	3.73882602	-3.50330187	-1.49176869
H	2.74843059	-4.09213904	-2.55306934
H	3.46848930	-3.77288166	0.70048324
H	3.84607780	-2.15505226	0.58195801
H	1.82098767	-2.78913587	-0.01899052
H	0.76110439	-3.41718670	-1.15212254
H	0.94476121	-5.27273958	-2.00285661
H	0.35158621	-4.23262370	-3.16019637
H	2.10903146	-6.72929333	-1.24732633
H	0.85935342	-6.38601432	-0.33721911
H	3.74677578	-5.75536158	0.50784751
H	2.55365106	-5.37830376	1.47865874
H	0.63670274	-4.47227248	0.78577195
H	0.05916988	-5.47304462	1.87882703
O	3.79198010	-6.43808567	-1.06709584

O	3.67839400	-4.15261433	-2.23124868
O	3.30889214	-2.86237591	0.18625813
O	0.81975393	-2.99451386	-0.20495111
O	0.91941528	-4.28206494	-2.37241206
O	1.13319590	-6.52190568	-1.27460880
O	3.47613868	-5.12871193	1.23597789
O	0.73844474	-5.37132039	1.19058549

ENERGY = -2106.22985600
ZPVE = 0.289434983368

Product

Fe	2.20819860	-0.00791760	0.07528077
C	4.22381438	-0.43122671	-1.24363411
C	4.17774632	1.36833399	0.41041943
C	4.01737036	-0.96905603	1.12537109
C	4.18443231	-1.38299023	-0.21150787
C	4.21888182	0.95301079	-0.93101229
C	4.01575513	0.41925098	1.43916953
H	4.28937671	-0.75466023	-2.28504674
H	4.20087490	2.43442767	0.65163116
H	3.95107774	-1.70937967	1.92569220
H	4.21274302	-2.44909447	-0.45207748
H	4.28106138	1.69307578	-1.73216653
H	3.94724831	0.74326986	2.48001207
H	-5.58036801	1.76612259	-0.14100006
H	-4.81495416	0.36301764	-0.37929033
H	-3.30753657	2.06199978	-0.85228624
H	-1.88676653	2.37947463	-0.24336656
H	-5.34654066	-1.76937395	-0.95909888
H	-3.75746980	-1.41774044	-1.23328333
H	-1.63759606	1.27281288	-1.91945263
H	-0.73254824	0.79366314	-3.18771032
H	-0.42916065	1.06224435	0.76967609
H	-0.48000675	2.37789819	1.74715733
H	-0.42647239	-1.25766733	0.30256576
H	-0.04069825	0.19622295	-0.97373923
H	-3.92144170	-1.61460349	0.84639824
H	-3.26560472	-0.39998090	1.95419379
H	-0.47411639	-2.85248137	0.68731131
H	-2.37452972	-1.84351065	1.27772308
H	-1.98294466	-0.91224071	-2.20090818
H	-1.88351024	-2.07743873	-1.13997168
H	-2.38234804	1.22170164	1.88493410
H	-3.93611801	1.17113109	1.42757066
O	-4.71853778	1.32043303	-0.06585676
O	-2.37890577	2.29882011	-1.09634031
O	-4.60681034	-1.22879440	-0.63014165
O	-1.06233340	0.50900066	-2.31832582
O	-1.03315456	1.77544876	1.22164676

O	0.29924070	0.01381298	-0.05310480
O	-3.28474741	-1.55741410	1.62532833
O	-1.03071327	-2.09341660	0.44404259
O	-2.47242183	-1.70314063	-1.84070066
O	-3.28576815	0.81860775	2.10480956

ENERGY = -2259.20441587
ZPVE = 0.351285721663

****** n = 10 cluster ***

$(C_2H_2)_2$-$Fe^+(H_2O)_{10}$

Fe	2.40226847	0.49840898	-0.10216181
C	3.04558497	1.29133177	-1.95569029
C	3.52904858	1.49365354	1.19013451
C	1.94770497	1.81587855	-1.71198679
C	3.10357828	0.43830113	1.75255764
H	3.98818389	1.05753369	-2.43861452
H	4.09805186	2.41967357	1.14467703
H	1.02184261	2.37988076	-1.76562945
H	3.05014678	-0.22935428	2.61103471
H	-4.74858644	2.16069199	-0.54624190
H	-4.18728805	0.65309248	-0.57515904
H	-2.39778500	2.12967192	-1.11623093
H	-1.01992293	2.15648493	-0.36118675
H	-5.12322916	-1.41505260	-0.84239633
H	-3.49216311	-1.43353026	-1.09337336
H	-0.99978871	0.84654475	-2.03356134
H	-0.23645560	0.08089979	-3.24736362
H	0.00614924	0.71484789	0.77739474
H	0.25900680	1.98968911	1.76616691
H	-0.13909075	-1.56241174	0.55610276
H	0.28783686	-0.38102108	-0.94946847
H	-3.70874359	-1.32404763	0.94808361
H	-2.97468740	-0.11786637	1.96939721
H	-0.46205174	-3.05254541	1.14670893
H	-2.21289411	-1.69851787	1.48967706
H	-1.69100651	-1.36340794	-2.06822773
H	-1.72177433	-2.35010220	-0.83137670
H	-1.89611425	1.37142523	1.83547350
H	-3.37701835	1.49338480	1.22367421
O	-3.95681777	1.61829436	-0.38186222
O	-1.41885731	2.13227126	-1.26640354
O	-4.29298208	-0.98735821	-0.56643445
O	-0.64123364	-0.05121629	-2.37301249
O	-0.40775643	1.56421843	1.19869075
O	0.67582235	-0.40052341	-0.03514160
O	-3.09722846	-1.27092288	1.75032825
O	-0.87468655	-2.24947723	0.78393237
O	-2.26328559	-2.02096210	-1.58993428

O	-2.85961219	1.13009821	2.00032218

ENERGY = -2181.70246086
ZPVE = 0.308948970867

Reactant

Fe	2.53742463	-0.03507293	0.05305452
C	3.45781569	-0.02957770	-1.83849597
C	2.99635837	1.68312938	1.19536137
C	2.84486862	-1.83678536	1.12535484
C	2.81903524	-2.12411309	-0.08624383
C	3.16506656	1.14912402	-1.55895083
C	2.58038138	0.81893283	1.99000320
H	3.86529683	-0.85069251	-2.41490025
H	3.40807191	2.62827823	0.86398150
H	2.95021762	-1.99384438	2.19146954
H	2.85747397	-2.74480410	-0.97299413
H	3.08571963	2.22101630	-1.69353724
H	2.29866522	0.38243728	2.94029130
H	-5.16101171	1.79349353	0.02614250
H	-4.41032438	0.38753439	-0.24215873
H	-2.94046038	2.08470024	-0.83742573
H	-1.49163268	2.44231027	-0.32987415
H	-4.98170901	-1.74899533	-0.75571094
H	-3.41203275	-1.40891546	-1.12865598
H	-1.34938776	1.27712084	-2.01729954
H	-0.55597552	0.77468659	-3.34895504
H	-0.03755937	1.11995675	0.60361654
H	0.07685288	2.41869232	1.59695978
H	-0.06628883	-1.22346088	0.18644177
H	0.26989888	0.20626239	-1.13843030
H	-3.44826170	-1.57298092	0.96556552
H	-2.71048942	-0.32835207	1.99857194
H	0.03104366	-2.80101947	0.61215381
H	-1.88497358	-1.82594497	1.30559864
H	-1.70296619	-0.92337282	-2.21738439
H	-1.53944998	-2.08843503	-1.15432648
H	-1.84506497	1.30277556	1.85961189
H	-3.42323682	1.21996667	1.49020711
O	-4.29523963	1.34977876	0.05097344
O	-2.03202713	2.32215106	-1.14762422
O	-4.22209458	-1.20649628	-0.47952056
O	-0.81404718	0.50651415	-2.45070174
O	-0.56045913	1.86486495	1.11558366
O	0.58867663	0.04742544	-0.20867778
O	-2.76338902	-1.50421500	1.69989739
O	-0.60107860	-2.09806763	0.38609753
O	-2.16857574	-1.70907263	-1.81610109
O	-2.72937059	0.88147932	2.13151631

ENERGY = -2258.99958638
ZPVE = 0.337524939104

Transition state (TS_{w10})

Fe	2.46196153	-0.15534808	0.06834441
C	3.27201251	0.28772047	-1.67220899
C	3.14534972	1.75873698	0.88464772
C	2.97155017	-1.82831476	1.09611760
C	3.11120821	-2.06317634	-0.13136218
C	3.47379875	1.40667204	-1.07504612
C	2.70068467	0.89708242	1.72696485
H	3.44848473	-0.19567325	-2.63108441
H	3.54814184	2.76874062	0.86469657
H	3.01406084	-2.05569892	2.15601991
H	3.37060633	-2.65860859	-1.00049630
H	3.89169124	2.38948971	-1.27906893
H	2.53928055	0.77777697	2.79679752
H	-5.14650786	1.86979379	0.01148492
H	-4.44355902	0.43656941	-0.23860659
H	-2.92713753	2.07064208	-0.87185730
H	-1.46883980	2.40546728	-0.37620124
H	-5.07507392	-1.69368956	-0.71723500
H	-3.50203682	-1.39644381	-1.11337631
H	-1.34864477	1.20737142	-2.03063783
H	-0.54605375	0.67041852	-3.34326757
H	-0.05001802	1.06976672	0.59714042
H	0.11627495	2.40325144	1.51766225
H	-0.13973161	-1.33668570	0.19364336
H	0.23567287	0.10204045	-1.14306693
H	-3.52595138	-1.53236557	0.97964990
H	-2.74465983	-0.31276216	1.99758057
H	-0.11543069	-2.92087038	0.59991827
H	-1.97065868	-1.83924968	1.32210241
H	-1.77792112	-0.97661411	-2.21639129
H	-1.65585848	-2.14057161	-1.14894959
H	-1.84497029	1.31364505	1.85571325
H	-3.41945411	1.26145999	1.48036628
O	-4.29569306	1.39812872	0.03960165
O	-2.01384215	2.27849350	-1.18959124
O	-4.29855991	-1.16760542	-0.45642418
O	-0.82539203	0.41717896	-2.44691012
O	-0.54368499	1.84080548	1.07842939
O	0.55210952	-0.06286074	-0.21051302
O	-2.83547889	-1.47767480	1.71188327
O	-0.70990711	-2.18000824	0.39233393
O	-2.27362885	-1.73935021	-1.80795936
O	-2.73463223	0.91001206	2.12352540

ENERGY = -2258.98228796

ZPVE = 0.338985929631

Product

Fe	2.20819860	-0.00791760	0.07528077
C	4.22381438	-0.43122671	-1.24363411
C	4.17774632	1.36833399	0.41041943
C	4.01737036	-0.96905603	1.12537109
C	4.18443231	-1.38299023	-0.21150787
C	4.21888182	0.95301079	-0.93101229
C	4.01575513	0.41925098	1.43916953
H	4.28937671	-0.75466023	-2.28504674
H	4.20087490	2.43442767	0.65163116
H	3.95107774	-1.70937967	1.92569220
H	4.21274302	-2.44909447	-0.45207748
H	4.28106138	1.69307578	-1.73216653
H	3.94724831	0.74326986	2.48001207
H	-5.58036801	1.76612259	-0.14100006
H	-4.81495416	0.36301764	-0.37929033
H	-3.30753657	2.06199978	-0.85228624
H	-1.88676653	2.37947463	-0.24336656
H	-5.34654066	-1.76937395	-0.95909888
H	-3.75746980	-1.41774044	-1.23328333
H	-1.63759606	1.27281288	-1.91945263
H	-0.73254824	0.79366314	-3.18771032
H	-0.42916065	1.06224435	0.76967609
H	-0.48000675	2.37789819	1.74715733
H	-0.42647239	-1.25766733	0.30256576
H	-0.04069825	0.19622295	-0.97373923
H	-3.92144170	-1.61460349	0.84639824
H	-3.26560472	-0.39998090	1.95419379
H	-0.47411639	-2.85248137	0.68731131
H	-2.37452972	-1.84351065	1.27772308
H	-1.98294466	-0.91224071	-2.20090818
H	-1.88351024	-2.07743873	-1.13997168
H	-2.38234804	1.22170164	1.88493410
H	-3.93611801	1.17113109	1.42757066
O	-4.71853778	1.32043303	-0.06585676
O	-2.37890577	2.29882011	-1.09634031
O	-4.60681034	-1.22879440	-0.63014165
O	-1.06233340	0.50900066	-2.31832582
O	-1.03315456	1.77544876	1.22164676
O	0.29924070	0.01381298	-0.05310480
O	-3.28474741	-1.55741410	1.62532833
O	-1.03071327	-2.09341660	0.44404259
O	-2.47242183	-1.70314063	-1.84070066
O	-3.28576815	0.81860775	2.10480956

ENERGY = -2259.20441587

ZPVE = 0.351285721663

****** n = 12 cluster **

Reactant

Fe	-2.21739249	3.74709680	2.41113672
C	-2.67599644	5.02299266	0.78222728
C	-3.98179162	2.73651786	3.01212452
C	-1.31143927	4.78107132	4.03259900
C	-0.72342272	5.12325657	2.99372069
C	-3.11932471	3.90241189	0.48312227
C	-3.15649352	2.56616576	3.92496093
H	-2.44581962	6.08004746	0.73243887
H	-4.90911538	2.71564240	2.45324182
H	-1.59498396	4.73433505	5.07629198
H	-0.02099707	5.61095875	2.32876022
H	-3.56027184	3.08079758	-0.07119405
H	-2.72762540	2.23506581	4.86281879
H	-0.01836207	2.02947342	2.09455339
H	-0.79287350	2.44218017	0.70383305
H	-1.97960164	0.91898842	1.67122627
H	-3.35453388	0.26778791	2.12997234
H	-2.16793597	-1.42836359	1.00847752
H	-1.89429222	-2.12351853	-0.37618298
H	-0.36539342	-2.53812550	0.97050059
H	0.81517962	-3.57215048	1.39415717
H	1.51584520	-1.48420052	1.56568550
H	2.62874862	-0.56913241	0.81044360
H	1.67149059	0.59241318	2.07407184
H	1.91094679	1.90884491	3.06545295
H	-0.85092684	3.33256755	-1.29232820
H	-1.38141318	1.79375674	-1.10445730
H	-2.20723458	0.01972670	-1.67519943
H	-2.79367496	0.58865166	-0.31912749
H	-1.76475754	-1.95040533	-2.75181739
H	-0.48519340	-1.50920965	-1.85273201
H	0.95657750	-2.33224301	-0.66505748
H	1.67077056	-1.15841836	-1.43904468
H	2.65052617	0.84863361	-0.74870342
H	3.81201962	-0.17406038	-1.23797200
H	1.12938567	2.30879607	-0.68887944
H	1.95307119	2.20579044	0.64280222
O	-0.95114464	2.28210386	1.70361243
O	-2.67535510	0.24298602	1.43514640
O	-1.84961499	-2.26762634	0.60454460
O	0.65226577	-2.67709901	1.05107519
O	2.15041507	-0.65283599	1.67939025
O	1.40056811	1.61814717	2.29065099
O	-0.64342718	2.49751027	-0.83843750
O	-2.50809180	0.86804787	-1.22187915
O	-1.51810146	-1.43827732	-1.96250145
O	0.93730818	-1.82203751	-1.51296890

O	2.93026674	-0.13183418	-0.83033615
O	2.04857682	2.22897117	-0.33935625

ENERGY = -2411.739928990000
ZPVE = 0.388778847609

Transition state

Fe	-2.02858749	3.73088249	2.52122055
C	-2.40384164	5.08569402	1.15000476
C	-4.14231776	3.18532535	2.52304819
C	-1.11445374	4.15663783	4.25988114
C	-0.70900570	4.97140095	3.38906360
C	-3.55443690	4.50607229	1.17475342
C	-3.42862450	2.75369237	3.50514580
H	-1.89491339	5.89844552	0.63461489
H	-5.16976369	3.11347165	2.17071790
H	-1.17429811	3.72398951	5.25337068
H	-0.13373657	5.81292897	3.01636940
H	-4.50376820	4.56638943	0.64591868
H	-3.53900422	2.15914014	4.41066926
H	-0.11266820	2.03732212	1.82086617
H	-1.03396960	2.48894833	0.48025067
H	-2.17696145	0.95420824	1.44054994
H	-3.62707937	0.38853819	1.75206839
H	-2.18144660	-1.40650862	1.03563845
H	-1.75721607	-2.22559168	-0.24493315
H	-0.30185521	-2.45529520	1.16370645
H	0.85915538	-3.43148291	1.75028263
H	1.63195176	-1.28493066	1.65097226
H	2.65076559	-0.41765025	0.76573941
H	1.64405404	0.71899703	1.92615885
H	1.69871180	2.16638563	2.80952548
H	-1.17045830	3.29817154	-1.52293556
H	-1.49831499	1.68055823	-1.32450915
H	-1.96888854	-0.28409166	-1.77727577
H	-2.81269386	0.42072484	-0.62597723
H	-1.57954907	-2.34314285	-2.61972471
H	-0.28327628	-1.86159599	-1.75649752
H	1.12710723	-2.48377442	-0.45230916
H	1.79000018	-1.37460195	-1.36239738
H	2.46520965	0.84252097	-0.92100853
H	3.74816342	-0.07200394	-1.30408833
H	0.87644965	2.32730245	-0.94816582
H	1.74585982	2.27487601	0.35846293
O	-1.09783783	2.29747485	1.50005990
O	-2.84512124	0.25942054	1.18979475
O	-1.74782780	-2.24257550	0.74800160
O	0.71519703	-2.59218056	1.28083324
O	2.21131700	-0.46429487	1.66095895
O	1.25709121	1.75164768	2.04924524

O	-0.89674913	2.51434212	-1.01645978
O	-2.38014557	0.59114907	-1.49539164
O	-1.31506043	-1.77196377	-1.87821611
O	1.13111692	-2.11626698	-1.37100162
O	2.86539952	-0.10060330	-0.89694454
O	1.80386031	2.21821053	-0.62431405

ENERGY = -2411.719241040000
ZPVE = 0.388809049242

Product

Fe	-2.04895747	3.50901342	2.33325691
C	-2.26058524	5.93669684	2.50165893
C	-4.23679151	4.51194842	2.72350539
C	-2.45619275	4.41214223	4.39738569
C	-1.76800836	5.44621343	3.71918311
C	-3.47853030	5.43731640	1.97616594
C	-3.72047353	3.97771154	3.91461547
H	-1.70024404	6.69613099	1.95082711
H	-5.20201112	4.16649829	2.34387828
H	-2.06440182	4.01594896	5.33657405
H	-0.82386012	5.82570801	4.11849867
H	-3.85971510	5.81669956	1.02555298
H	-4.29088871	3.23263590	4.47439026
H	0.20573471	1.75545180	1.73393350
H	-1.07287780	2.32706708	0.39193226
H	-1.97741683	0.78474316	1.30682181
H	-3.34753265	-0.02794723	1.60071172
H	-1.78748825	-1.58638729	0.96780166
H	-1.45221199	-2.49884129	-0.29732916
H	-0.02437643	-2.62598166	1.02930423
H	1.21000973	-3.52504619	1.73195231
H	1.78043755	-1.57877005	1.41342899
H	2.74787113	-0.53303857	0.46628600
H	1.85444075	0.16627854	1.69959934
H	1.56381812	2.05725330	2.62707026
H	-1.10825747	2.99838773	-1.86355164
H	-1.34824114	1.42123634	-1.51577935
H	-1.82550362	-0.62841835	-1.88977392
H	-2.56509476	0.08095469	-0.68417840
H	-1.44641093	-2.79637055	-2.64880396
H	-0.11446512	-2.23432549	-1.91630831
H	1.42298425	-2.78117500	-0.66095460
H	1.96364214	-1.61356646	-1.56506489
H	2.46280196	0.72011597	-1.06303253
H	3.82964838	-0.15161974	-1.37282289
H	0.86789937	2.12253755	-1.13808093
H	1.73367489	2.08436611	0.17994463
O	-1.15211148	2.12306916	1.36854210
O	-2.51378042	-0.06266449	1.10252619

O	-1.36313798	-2.44465114	0.69414966
O	1.03915773	-2.75369281	1.16410760
O	2.37181750	-0.68606929	1.42175823
O	1.23353055	1.54215078	1.87146259
O	-0.79920741	2.28244399	-1.28249159
O	-2.22170407	0.23722543	-1.59735722
O	-1.14077560	-2.18461815	-1.95705066
O	1.37304042	-2.40634974	-1.56996514
O	2.92911915	-0.22299321	-1.00989420
O	1.79996945	1.99831808	-0.79994504

ENERGY = -2411.943831140000
ZPVE = 0.401031316184

****** n = 18 cluster **

Reactant

Fe	0.34803279	0.35890923	2.72496928
C	-0.12987242	2.45379293	2.72588707
C	-1.08238537	-0.98576087	3.43571127
C	2.40094366	-0.09562566	2.86070844
C	2.31281023	1.14124588	2.94962497
C	-1.12708112	1.83832862	3.13388002
C	0.02928610	-1.35733121	3.86489761
H	0.49247928	3.28469693	2.42477058
H	-2.15353586	-1.00468150	3.26323151
H	2.84326998	-1.08200745	2.80212971
H	2.62670048	2.16731613	3.08423932
H	-2.12358713	1.65262871	3.51073388
H	0.74976845	-1.94907321	4.41732909
O	-0.18729670	-0.41512457	-2.05444850
H	0.74172473	-0.75285197	-2.42441511
H	0.22672565	1.21600135	-2.82263370
O	-1.47602254	-2.83811740	-1.93259522
H	-1.30883847	-3.08269826	-0.98969864
H	-0.97703089	-1.98836939	-2.04756487
O	2.11416060	-1.14401940	-2.74169951
H	2.46285420	-1.14052859	-1.80717196
H	2.56119909	-0.33805460	-3.17004079
O	0.43084684	2.18235483	-2.84266145
H	-0.97671893	2.86121859	-2.90994744
H	0.74115293	2.34318393	-1.90425755
O	-1.93400236	3.11458430	-2.60065610
H	-2.45814866	2.25025711	-2.62313229
H	-1.79280625	3.39668810	-1.54558370
O	-2.64302147	0.61603695	-2.25369171
H	-1.75990142	0.18641852	-2.40688690
H	-3.30981925	-0.12968489	-2.28147166
O	-3.85494348	-1.72046273	-1.84630112
H	-3.03097146	-2.29441160	-1.98743095

H	-4.60530475	-2.14152921	-2.30195623
O	1.25562333	-3.74473696	-2.39137259
H	0.29399638	-3.72742275	-2.57417031
H	1.65303640	-2.98166724	-2.86792325
O	3.07387484	1.20543332	-3.30201246
H	3.41781912	1.45681382	-2.41349212
H	2.32227690	1.80726614	-3.48459620
O	0.09020691	-0.11362413	0.74629194
H	-0.22715211	-1.05071515	0.78093382
H	-0.05490093	-0.24863832	-1.08554826
O	-1.10528922	-2.63368601	0.77380173
H	-2.03076829	-2.16037465	0.81269387
H	-1.08737322	-3.23814701	1.53761163
O	2.59641184	-0.89909233	-0.08795787
H	1.73341769	-0.53543802	0.24119084
H	2.48734625	-1.90220706	0.09140522
O	1.06318833	2.29354406	-0.23142931
H	2.05904035	2.19636828	-0.21950079
H	0.71680223	1.43957665	0.11995958
O	-1.44384710	3.50832707	-0.20535444
H	-1.87516911	2.73322528	0.26020705
H	-0.47198134	3.38094133	-0.09108966
O	-2.31450816	1.06123350	0.38747229
H	-2.50430871	0.91088378	-0.58196209
H	-1.41510307	0.65363058	0.55037813
O	-3.33695024	-1.40632397	0.82236833
H	-3.79736415	-1.58257974	-0.03833153
H	-3.17182474	-0.42725054	0.83759040
O	1.75694808	-3.30734554	0.16394461
H	0.83661000	-3.16627421	0.45369632
H	1.66001521	-3.63439428	-0.78488227
O	3.58002702	1.47561647	-0.53781634
H	4.43442521	1.77923313	-0.18512471
H	3.46427225	0.50725872	-0.29683224

ENERGY = -2869.984037880000
ZPVE = 0.546276925108

Transition state

Fe	0.47803914	0.23783051	2.62345703
C	0.38620081	2.20112126	2.72575418
C	-1.47609106	0.12752096	3.59932467
C	2.06397207	-0.93881966	2.98382336
C	2.37454520	0.23961676	3.29449071
C	-0.80332730	1.93062408	3.13831994
C	-0.79221596	-0.95948592	3.53108853
H	0.99712184	3.07633924	2.51521020
H	-2.46021555	0.44979208	3.93243781
H	2.21317016	-2.00373484	2.82486140
H	3.02089864	1.03171479	3.65588035

H	-1.70645521	2.46177148	3.43086800
H	-0.85204708	-2.00562098	3.82055023
O	-0.17509151	-0.38020170	-2.12915389
H	0.76307900	-0.70540132	-2.49093458
H	0.12732020	1.26269686	-2.91848402
O	-1.35864177	-2.85359963	-1.91485042
H	-1.18780118	-3.00637630	-0.94956542
H	-0.90181597	-1.99392999	-2.10162619
O	2.14843425	-1.07258005	-2.78011282
H	2.50809621	-1.01077497	-1.85016061
H	2.54799368	-0.26523468	-3.24562552
O	0.28397361	2.23815076	-2.93988596
H	-1.14609018	2.86424208	-2.91288685
H	0.61676390	2.40974064	-2.01168033
O	-2.09702902	3.07784547	-2.55458630
H	-2.58605737	2.19384776	-2.56404721
H	-1.93354944	3.35981862	-1.49936240
O	-2.68392139	0.55015970	-2.19272408
H	-1.78979019	0.16627421	-2.39331058
H	-3.31666545	-0.22409915	-2.23501883
O	-3.77666966	-1.84167998	-1.83864332
H	-2.92940978	-2.38224915	-1.98397216
H	-4.51697770	-2.29860089	-2.27558693
O	1.42801943	-3.66020838	-2.09510934
H	0.47671051	-3.72264606	-2.31885670
H	1.80447897	-2.95299913	-2.66455042
O	2.96106972	1.32092452	-3.41711460
H	3.30684406	1.62128633	-2.54485665
H	2.18929228	1.89079053	-3.61541337
O	0.13799453	-0.12003977	0.66717631
H	-0.15385105	-1.06272051	0.70265453
H	-0.04258946	-0.21060332	-1.16251263
O	-1.02452644	-2.67474588	0.78723931
H	-1.96806675	-2.22508032	0.87137219
H	-1.03321934	-3.45615152	1.36813322
O	2.68325672	-0.68252444	-0.17211366
H	1.78892090	-0.38491504	0.14013826
H	2.68690107	-1.64079320	0.15323701
O	0.96420037	2.38949556	-0.34946472
H	1.96436398	2.34860360	-0.33767544
H	0.66018862	1.53730488	0.03445059
O	-1.57522554	3.48013321	-0.16804166
H	-1.94954570	2.67303142	0.29410654
H	-0.59459157	3.41603473	-0.07338818
O	-2.32664670	1.00235781	0.44963539
H	-2.53816794	0.83788278	-0.51314094
H	-1.42012043	0.60957626	0.58320812
O	-3.27985632	-1.53591824	0.83147307
H	-3.69956538	-1.68038731	-0.05633075
H	-3.16533197	-0.55804886	0.92834312

O	1.92637869	-3.09141684	0.45000165
H	0.99687418	-2.91410931	0.68746395
H	1.85019905	-3.48073056	-0.47459868
O	3.52161295	1.73014287	-0.67921077
H	4.36507275	2.10650063	-0.37321883
H	3.48036333	0.76544069	-0.40453521

ENERGY = -2869.964012740000
ZPVE = 0.547213663145

Product

Fe	0.91750691	0.09804726	1.99949563
C	1.56266712	1.60183947	3.55443809
C	-0.80613839	0.98683129	3.38143930
C	0.84394185	-0.66338526	4.13941384
C	1.84036516	0.33245743	4.10443444
C	0.22139356	1.93010378	3.18889723
C	-0.49329668	-0.33281620	3.76403625
H	2.34931976	2.35568000	3.48065446
H	-1.83291399	1.23983668	3.10299847
H	1.07721358	-1.66356522	4.51066895
H	2.86209003	0.08508087	4.40623716
H	-0.01241257	2.92922432	2.81490712
H	-1.28287807	-1.08447462	3.83529823
O	-0.20746420	-0.32402394	-2.41669924
H	0.72001853	-0.68033765	-2.61039338
H	0.03664475	1.33263894	-2.99421121
O	-1.37350386	-2.83902523	-2.19121044
H	-1.20041983	-2.86735969	-1.20516922
H	-0.99265313	-1.96691124	-2.45870527
O	2.30682790	-1.06451614	-2.59798042
H	2.66444211	-1.09780384	-1.68831300
H	2.65257522	-0.18442450	-2.97060457
O	0.14291613	2.32052990	-2.98859759
H	-1.34342229	2.84158339	-2.97262022
H	0.41223063	2.50918342	-2.04512097
O	-2.31180526	2.99272803	-2.63382714
H	-2.76842680	2.09504089	-2.67348743
H	-2.21269054	3.27345966	-1.55801713
O	-2.83748233	0.43528423	-2.31077150
H	-1.95388035	0.09685529	-2.59192633
H	-3.44143060	-0.36405924	-2.30528422
O	-3.84614441	-1.97790567	-1.85875827
H	-3.00135876	-2.47642971	-2.10371491
H	-4.60211864	-2.44311957	-2.25883159
O	1.33174849	-3.59882109	-2.02474147
H	0.39036679	-3.62356969	-2.31516136
H	1.78590900	-2.92434496	-2.57611050
O	2.90011438	1.42042358	-3.05921193
H	3.11811605	1.72750994	-2.15016528

H	2.14150543	1.95883339	-3.36980517
O	-0.05567651	-0.08594480	0.19636757
H	-0.42642013	-1.01849758	0.27898094
H	-0.17749010	-0.16276472	-1.42024635
O	-0.94199772	-2.53128109	0.39293878
H	-1.86701177	-2.40681039	0.81716570
H	-0.27557894	-3.06520801	0.87521535
O	2.40810060	-0.75102814	0.41948723
H	1.51307437	-0.55946984	0.00324139
H	2.38191893	-1.75324002	0.56541601
O	0.60639518	2.48789158	-0.34481243
H	1.58231249	2.47006175	-0.17090430
H	0.29370117	1.57880968	-0.11186086
O	-1.95096497	3.40968429	-0.22930942
H	-2.29608237	2.57822820	0.21649926
H	-0.97109324	3.40083651	-0.09355918
O	-2.54219133	0.88486510	0.38276091
H	-2.77897152	0.71700429	-0.56899517
H	-1.59698672	0.56356902	0.42903787
O	-3.30585611	-1.77237629	0.89519648
H	-3.72370083	-1.93343330	0.01393699
H	-3.24101662	-0.79013415	0.96692162
O	1.70868558	-3.27446318	0.51874453
H	2.21697746	-4.01969554	0.88428354
H	1.57021245	-3.46857192	-0.47767699
O	3.21918184	1.73941189	-0.26461479
H	4.01959927	2.15705063	0.09952167
H	3.20055154	0.79650541	0.04634779

ENERGY = -2870.192951400000
ZPVE = 0.559612175071
